# Bridging the Knowledge Gap: Awareness, Attitudes, and Practices Regarding Menstrual Cup Usage Among Medical Students in Chennai, India

**DOI:** 10.7759/cureus.82780

**Published:** 2025-04-22

**Authors:** Swathika Devi R, Anantha Eashwar V M, Sujitha Pandian, Monica Albert Sekhar, Sushmitha G, Kiruthika Narayanan, Gowtham S, Nikhil C M

**Affiliations:** 1 Community Medicine, Sree Balaji Medical College and Hospital, Chennai, IND

**Keywords:** behavioural patterns, health perception, hygiene practices, menstrual health, sustainable menstrual products

## Abstract

Background

In India, sanitary pads are the most used menstrual hygiene product despite potential health and environmental risks. Menstrual cups offer a safer, sustainable alternative, but lack widespread study. This study examines awareness, attitudes, and practices of menstrual cup use among medical students and factors influencing adoption.

Methods

This cross-sectional study was conducted at a private medical college in Chennai in India among 250 medical students. Participants were selected through simple random sampling. After obtaining informed consent, data collection was carried out using a pre-structured and pre-tested questionnaire. Data was entered into MS Excel (Microsoft® Corp., Redmond, WA, USA) and analyzed using IBM SPSS Statistics for Windows, Version 25 (Released 2017; IBM Corp., Armonk, New York, United States). Descriptive statistics are presented in tables, analytical statistics include the calculation of unadjusted odds ratios, followed by logistic regression analysis to assess associations between relevant variables.

Results

Among the 250 study participants, more than two-thirds were aged between 18 and ≤ 21 years. Poor knowledge about menstrual cups (56.4%), unfavorable attitudes (57.6%), and menstrual cup usage of 46.4% were noted among the study participants. Key factors significantly associated with poor knowledge about menstrual cups included family members who did not use menstrual cups (adjusted odds ratio (AOR) 3.21), non-availability of menstrual cups (AOR 5.12), experiencing frequent menstrual problems (AOR 2.11), and receiving doctors’ advice regarding menstrual issues (AOR 2.91). Unfavorable attitudes were linked to concerns while purchasing sanitary napkins (AOR 3.60), non-usage by family members (AOR 2.16), non-availability (AOR 3.10), menstrual issues (menorrhagia, recurrent infections), disposal concerns (AOR 2.60), and perceived difficulty of use (AOR 4.17).

Conclusion

This study highlights the necessity for enhanced education on menstrual hygiene practices, even within the medical community, to address the knowledge gap. It is essential to educate young medical students on the benefits and drawbacks of menstrual cups, with a focus on why they are a safer and more eco-sustainable option. Future research should be conducted in community settings to allow for the generalization of the findings.

## Introduction

In 2022, the World Health Organization (WHO) urged that menstrual health be defined and treated as a human right and health issue, not merely a hygiene one. Globally, the WHO demands three actions: (a) Menstruation should be viewed as a health issue with physical, psychological, and social dimensions. (b) To understand that menstrual health entails that menstruating individuals have access to information and education about menstruation, various available and suitable menstrual products, water, sanitation, and disposal facilities, an environment where menstruation is viewed as a healthy, positive physiological process, and the ability to fully engage in social and professional activities. (c) To ensure menstrual health activities are included in sectoral plans and budgets, and their performance is to be measured. WHO aims to break menstrual stigma and create menstruation-responsive environments in schools, health facilities, and various other workplaces [1].

In India, the current population is about 1.42 billion, among which 22.2% are women belonging to the reproductive age group (15-49 years) [2]. During menstruation, these women follow various menstrual hygiene practices, including the usage of sanitary pads, tampons, menstrual cups, and cloths. Annually, on average, a woman throws away around 150 kg of non-biodegradable waste generated during menstruation. This adds up to dumping in landfills, and improper handling of these can pose a major threat to land, oceans, and humankind. Sanitary pads and tampons take 500-800 years to decompose completely in a natural environment [3].

Around 90% of the material used in sanitary napkins is polypropylene, polyethylene, and superabsorbent polymers, which are non-biodegradable [4]. The majority of women utilize locally made sanitary pads, which often contain potentially fatal chemicals like bisphenol A and bisphenol S that interfere with foetal development, because company-made sanitary pads are not affordable for women. Also, cervical cancer may be brought on by the fibres used in the pads [5]. Ovarian cancer may also be caused by dioxin, which is found in certain menstrual pads [6]. At times, these are flushed down the toilets, causing major plumbing issues in the sewer systems and at wastewater treatment plants [7].

Reusable menstrual care products like menstrual underwear/pads and menstrual cups are better alternatives to disposables since these have environmental sustainability and cost sustainability [8,9]. The safe alternative to these is the use of menstrual cups among the reproductive age group. Menstrual cup is made up of medical-grade silicone, latex, and thermoplastic elastomers, which are non-toxic and non-allergic [10,11]. The insertion of menstrual cups is similar to tampons. Once it is inserted into the vagina, high up to the cervix, the menstrual cups have receptacles, which collects the menstrual flow [12]. The cups can be used for up to 12 hours and later emptied, depending on the individual’s menstrual flow. Cups can be reused for further cycles and can last up to 10 years [12]. The material used is resistant to the growth of bacteria and hence doesn’t cause urogenital infection. It comes in different sizes, shapes, and firmnesses, based on the material used for comfortable and safe usage. Hence, the menstrual cup has a greater advantage than other menstrual care products, but it has limited usage among women of the reproductive age group. Owing to less awareness and advertisements, these are not widely used by menstruating women [13,14].

With a view of the above background, this study is conducted to assess the awareness, attitude, and practices regarding menstrual cup usage among female medical students of a private medical college, and also to determine the factors associated with it.

## Materials and methods

Study design

The present study was a cross‐sectional study conducted among the medical students of Sree Balaji Medical College and Hospital, Chennai, India. The study was conducted between the months of December 2023 and May 2024. The study included all the female medical students between the age group of 18-25 years, who knew about menstrual cups. Female students who were not willing to participate and who were chronic absentees were excluded.

Sample size

Based on a study conducted by Brindhaavanan et al., where the prevalence of knowledge about menstrual cup usage was found to be 80%, the sample size was calculated accordingly [13]. The sample size (n) was determined using the formula:

\[ n = \frac{Z^2 \cdot P \cdot Q}{L^2} \]

where Z = 1.96 at a 95% confidence interval, P = prevalence, Q = (1 - P), and L = allowable error.

Substituting the values:

\[ n = \frac{1.96 \times 1.96 \times 80 \times 20}{5 \times 5} = 245 \]

Rounding off, the minimum required sample size was finalized at 250.

Sampling method

The total number of undergraduate medical students is 750, of whom 400 are female undergraduate medical students. From the records maintained in the medical college, the names of the female students were arranged alphabetically and then assigned a chronological number. By a random number generator, 250 random numbers were chosen. The student corresponding to the number was selected and included in the study.

Study tools

A predesigned, pretested semi-structured questionnaire was used (see Appendix A), and data were collected by face-to-face interviews. The questionnaire contained details about socio-demographic characteristics, questions related to their menstrual health and history, and questions on knowledge, attitude, and practice of the menstrual cup.

Knowledge was measured quantitatively using questions like duration of usage, do cups need to be sterilized before the next cycle, the correct way to remove cups, the material the cups are made of, etc., and a score ranging from 6 to 12 was given. Attitude was measured using questions like using cups is more convenient, cost-effective, usage can produce odour, recommendation to others, and a score ranging from 6 to 12 was given. Those who were currently using menstrual cups were considered to be practicing menstrual cups.

For knowledge, the mean score was found to be 8 ± 2 SD. All those who scored above the mean score were taken to have good knowledge about the menstrual cup. For attitude, the mean score was found to be 7 ± 1.9 SD. All those who scored above the mean score were taken to have a favourable attitude towards the menstrual cup. The questions were validated using Cronbach’s alpha, and the reliability coefficient was found to be 0.82 for the knowledge domain and 0.7 for the attitude domain. Face validity of the questions was assessed by discussion with experts in the field of gynaecology and women’s health.

Data analysis

Data was analyzed using IBM SPSS Statistics for Windows, Version 25 (Released 2017; IBM Corp., Armonk, New York, United States). The dependent variables, which were found to be significant in bivariate analysis at a 95% confidence interval, were included for logistic regression.

## Results

Table 1 shows the distribution of socio-demographic data and other variables related to menstrual cup usage. More than two-thirds of the study participants were between the ages of 18 and ≤ 21 years. Seventy-six percent of the participants’ family members in their household and their friends use menstrual cups.

**Table 1:** Socio-demographic data and variables related to menstrual cup usage among the study participants * Socio-economic status (SES) was classified using the Modified Kuppuswamy Scale.

Variables	Category	Frequency (N = 250)	Percentage (%)
Age	18 to ≤ 21 years	182	72.8
	≥ 22 to 25 years	68	27.2
Marital status	Married	15	6
	Unmarried	235	94
Socio-economic status (SES)*	Upper	149	59.6
	Upper middle	81	32.4
	Lower middle	20	8
How did you know about the usage of menstrual cup?	Family member	11	4.4
	Friend	29	11.6
	Social media	210	84
What is the menstrual cup made of?	Silicone	120	48
	Rubber/Latex	64	25.6
	Plastic	5	2
	Don’t know	61	24.4
Menstrual cup usage among family members	Yes	60	24
	No	190	76
Menstrual cup usage among friends	Yes	192	6.8
	No	58	23.2
Availability of menstrual cups in stores	Yes	104	42.6
	No	146	58.4
Menstrual problems (menorrhagia, recurrent infections)	Yes	111	44.4
	No	139	55.6
Buying sanitary napkins is a concern	Yes	99	39.6
	No	151	60.4
Advised by a doctor on problems related to menstrual hygiene	Yes	87	34.8
	No	163	65.2
Do you engage in sports activities?	Yes	93	37.2
	No	157	62.8
Do you feel menstrual cups are difficult to use?	Yes	100	40
	No	150	60
Disposal of sanitary napkins is a concern	Yes	115	46
	No	135	54

Table 2 shows that the study had a slightly greater proportion of individuals with poor knowledge (56.4%) and unfavourable attitudes (57.6%) toward menstrual cups. However, there is still a sizable portion (46.4%) who use menstrual cups.

**Table 2:** Knowledge, attitude, and practice of study participants regarding menstrual cup

Variable	Frequency (N = 250)	Percentage (%)
Good knowledge	109	43.6
Poor knowledge	141	56.4
Favourable attitude	106	42.4
Unfavourable attitude	144	57.6
Currently using menstrual cups	116	46.4

Table 3 shows the association between knowledge regarding menstrual cups and related variables. Those who had poor knowledge had higher odds of having family members who don't use menstrual cups, non-availability of menstrual cups, who had menstrual problems frequently, and individuals who were not advised by a doctor regarding menstrual problems were found to be statistically significant.

**Table 3:** Association between knowledge regarding menstrual cups and related variables Chi-square test, odds ratio, and logistic regression were used to test the association at a 95% confidence interval (CI). * p-value obtained from bivariate analysis, statistically significant at p < 0.05. ^†^ 95% CI: 95% confidence interval ** p-value obtained from logistic regression analysis, statistically significant at p < 0.05.

S. No.	Variables	Category	Poor knowledge	Good knowledge	Chi-square (χ²)	p-value*	Unadjusted odds ratio (95% CI)^†^	p-value**	Adjusted odds ratio (95% CI)
			Frequency (%)	Frequency (%)					
1	Age	18 to ≤ 21 years	99 (54.4)	83 (45.6)	1.09	0.29	0.73 (0.41-1.30)	-	
		≥ 22 to 25 years	42 (61.8)	26 (38.2)					
2	Menstrual cup usage among family members	No	52 (86.7)	8 (13.3)	29.41	0.00*	7.37 (3.32-16.36)	0.002**	3.21 (1.12-5.64)
		Yes	89 (46.8)	101 (53.2)					
3	Menstrual cup usage among friends	No	50 (86.2)	8 (13.8)	27.28	0.00*	6.93 (3.12-15.41)	0.87	1.11 (0.6-1.78)
		Yes	91 (47.4)	101 (52.6)					
4	Availability of menstrual cups in stores	No	104 (71.2)	42 (28.8)	31.4	0.00*	4.48 (2.61-7.68)	0.001**	5.12 (3.12-7.85)
		Yes	37 (35.6)	67 (64.4)					
5	Menstrual problems	No	101 (72.7)	38 (27.3)	33.66	0.00*	4.71 (2.75-8.07)	0.000**	2.11 (1.24-3.98)
		Yes	40 (36)	71 (64%)					
6	Buying sanitary napkins is a concern	Yes	74 (74.7)	25 (25.3%)	22.43	0.00*	3.71 (2.12-6.46)	0.95	0.8 (0.23-1.24)
		No	67 (44.4)	84 (55.6)					
7	Disposal of sanitary napkins is a concern	No	95 (70.4)	40 (29.6)	23.29	0.00*	3.56 (2.10-6.02)	0.88	0.95 (0.23-1.56)
		Yes	46 (40)	69 (60)					
8	Advised by a doctor on problems related to menstrual hygiene	No	122 (81)	31 (19)	115.09	0.00*	36.90 (16.69-81.57)	0.021**	2.91 (1.14-7.25)
		Yes	9 (10.3)	78 (89.7)					
9	Do you engage in sports activities?	No	86 (54.8)	71 (45.2)	0.45	0.50	0.83 (0.49-1.40)	-	
		Yes	55 (59.1)	38 (40.9)					
10	Do you feel menstrual cups are difficult to use?	Yes	56 (56)	44 (44)	0.01	0.91	0.97 (0.58-1.62)	-	
		No	85 (56.7)	65 (43.3)					

Table 4 shows the association between attitude regarding menstrual cups and its related variables. Those who had an unfavourable attitude had higher odds of having concerns while buying sanitary napkins, non-usage of menstrual cups among family members, non-availability of menstrual cups, menstrual problems experienced, concerns regarding disposal of sanitary napkins, and feeling that menstrual cups are difficult to use were also found to be significant.

**Table 4:** Association between attitude regarding menstrual cups and related variables Chi-square test, odds ratio, and logistic regression were used to test the association at a 95% CI. * p-value obtained from bivariate analysis, statistically significant at p < 0.05. ^†^ 95% CI: 95% confidence interval ** p-value obtained from logistic regression analysis, statistically significant at p < 0.05.

S. No.	Variables	Category	Unfavourable attitude	Favourable attitude	Chi-square (χ²)	p-value*	Unadjusted odds ratio (95% CI)^†^	p-value**	Adjusted odds ratio (95% CI)
			Frequency (%)	Frequency(%)					
1	Age	18 to ≤ 21 years	112 (61.5)	70 (38.5)	4.25	0.03*	1.80 (1.026-3.15)	-	
		≥ 22 to 25 years	32 (47.1)	36 (52.9)					
2	Menstrual cup usage among family members	No	47 (78.3)	13 (21.7)	13.89	0.00*	3.46 (1.76-6.82)	0.032**	2.16 (1.05-4.79)
		Yes	97 (51.1)	93 (48.9)					
3	Menstrual cup usage among friends	No	46 (79.3)	12 (20.7)	14.57	0.00*	3.67 (1.83-7.37)	0.069	1.67 (0.44-1.89)
		Yes	98 (51)	94 (49)					
4	Availability of menstrual cups in stores	No	111 (76)	35 (24)	48.79	0.00*	6.82 (3.89-11.95)	0.004**	3.10 (1.26-7.26)
		Yes	33 (31.7)	71 (68.3)					
5	Menstrual problems	No	104 (74.8)	35 (25.2)	38.01	0.00*	5.27 (3.05-9.09)	0.233	1.62 (0.64-4.02)
		Yes	40 (36)	71 (64)					
6	Buying sanitary napkins is a concern	Yes	71 (71.7)	28 (28.3)	13.37	0.00*	2.70 (1.57-4.65)	0.002**	3.60 (1.66-7.81)
		No	73 (48.3)	78 (51.7)					
7	Disposal of sanitary napkins is a concern	No	91 (67.4)	44 (32.6)	11.55	0.00*	2.41 (1.44-4.04)	0.001**	2.606 (1.06-6.40)
		Yes	53 (46.1)	62 (53.9)					
8	Advised by a doctor on problems related to menstrual hygiene	No	82 (50.3)	81 (49.7)	10.20	0.00*	0.40 (0.23-0.71)	0.301	2.11 (0.77-4.61)
		Yes	62 (71.3)	25 (28.7)					
9	Do you engage in sports activities?	No	124 (79)	33 (21)	78.99	0.00*	13.71 (7.33-25.65)	0.243	1.69 (0.69-4.12)
		Yes	20 (21.5)	73 (78.5)					
10	Do you feel menstrual cups are difficult to use?	Yes	72 (72)	28 (28)	14.15	0.00*	2.7 (1.62-4.78)	0.001**	4.17 (1.80-9.64)
		No	72 (48)	78 (52)					

Table 5 gives information regarding the practices of menstrual cups among the 116 participants who used menstrual cups. Consistent leakage was experienced by 7.8% of participants. A vast majority of participants experienced no side effects.

**Table 5:** Menstrual cup practices among medical students UTI: urinary tract infection

S. No.	Questions regarding practice	Yes (%), N = 116
1	What sanitary product were you using before menstrual cup?	
	Tampons	5 (4.3)
	Sanitary pads	107 (92.2)
	Homemade cloths	4 (3.4)
2	How do you store the menstrual cup?	
	Cloth bags	40 (34.5)
	Plastic bags	27 (23.3)
	Air-tight containers	49 (42.2)
3	Have you experienced any leakage?	
	Yes	9 (7.8)
	Sometimes	47 (40.5)
	No	60 (51.7)
4	Have you encountered any side effects?	
	Pain	28 (24.1)
	Rashes/dryness	6 (5.2)
	UTI	10 (8.6)
	None	72 (62.1)
5	After each use, do you wash the menstrual cup?	
	Yes	95 (81.9)
	Sometimes	8 (6.9)
	No	13 (11.2)
6	How often do you have to empty?	
	Once/day	27 (23.3)
	2-4 times/day	73 (62.9)
	>5 times/day	16 (13.8)

## Discussion

In the present study, good knowledge regarding menstrual cup usage was seen in 43.6% of the study participants. A study done in a medical institution in Mangalore by Ballal and Bhandary had a prevalence of good knowledge about menstrual cups of 65.6% [14]. Similar results were found in studies done by Shanmugham et al., which reported good knowledge at 61.2% [15]. This difference may be because it included participants from the age group of 16 to over 40 years, and from both medical and paramedical students and faculty members. Madi et al., in a study done in Karnataka, found that 51.6% of study participants had adequate knowledge regarding menstrual cups [16]. Although awareness about menstrual cups was high among medical students in the current study, in-depth knowledge of the use and type of material used was poorly understood even within the medical community. This finding was also similar to other studies done in India [15,17]. The current study showed that social media content on menstrual cups was the source of information on menstrual cups. This finding was similar to a study done by Sudevan et al., where 76.6% of participants got their information from social media [10]. This shows that social media has been a large source of information, and not medical colleges per se, showing the lack of menstrual health education across educational platforms. In the present study, 48% of students were aware that menstrual cups are made of silicone. In comparison, a study conducted by Eti et al. in 2019 among 400 undergraduate students reported a lower awareness level of 28% [18]. This difference may be attributed to the increasing influence and penetration of social media in recent years, particularly following the COVID-19 pandemic. However, recent studies done by Brindhaavanan et al. and Arumadi et al. showed that 51% and 59.6% of students, respectively, knew that menstrual cups were made of silicone [13,19].

An unfavourable attitude was seen among 144 people (57.6% of the population). A study done among nursing, dental, and engineering students showed a negative attitude of 4.5% regarding menstrual cup usage [20]. A total of 91.7% had a favourable attitude towards menstrual cups in a study done in North Kerala [19]. A little less than half of the study population agreed that menstrual cups were more cost-effective, and 34% agreed that menstrual cups can be worn overnight. Brindhaavanan et al. found a higher percentage of students agreeing that menstrual cups can be worn overnight [13]. About 22% of participants felt that menstrual cups did not produce odour. Other studies showed a higher percentage of the population not reporting odour with menstrual cup usage [13,21,22].

The current study showed that 46% of study participants were using menstrual cups. Whereas a similar finding of 42% menstrual cup usage was found in a study done by Jafrin et al. among the medical students in a private college in Pondicherry [23]. A study done in Kancheepuram district in a tertiary care centre by Shanmugham et al. showed that only 4.4% of doctors were using menstrual cups [15]. Similarly, a lower percentage of menstrual cup usage was seen in other studies done among reproductive-age women [14]. A study done in Kerala showed that 15% had tried using a menstrual cup [10]. The varying findings in this study show that usage may be affected by various cultural beliefs, upbringing, and exposure to menstrual cup awareness. The higher usage percentage in our study may be attributed to the urban setting and the social media influence during the COVID-19 pandemic years.

Regarding knowledge, it was found that the availability of menstrual cups, menstrual health problems, and being advised by a doctor on problems related to menstrual hygiene were variables that were found to be significantly associated with knowledge. Medical advice recommending menstrual cups as an alternative to other menstrual hygiene products, particularly in the context of recurrent infections or rashes, may significantly enhance participants' knowledge and have a more positive attitude towards their usage. Madi et al. found that the availability of menstrual cups had an association with good knowledge about menstrual cups [16]. Similar findings were also seen in other studies done by Sudevan et al., which was done among females in Southern Kerala [10].

Regarding attitude, the variables found to be statistically significant included menstrual cup usage among family members, availability of menstrual cups, concerns about purchasing sanitary napkins, and concerns related to the disposal of sanitary napkins. A study done among reproductive women in Turkey by Balkan et al. showed 42.9% had difficulty in using it [24]. Although no studies found an association between concerns regarding buying and disposing of sanitary napkins, a study in Kerala showed that the financial burden of purchasing disposable sanitary napkins was a concern [10]. Concerns regarding disposal were also highlighted in studies conducted by Kattimani et al., which may be one of the reasons for the shift towards using menstrual cups [3]. Multiple studies showed that feeling that menstrual cups were difficult to use and fear of insertion as the most common reasons for not using menstrual cups.

Menstrual hygiene is one of the most important factors that often goes overlooked in our community due to the lack of awareness and attitude among women. The findings highlight the important fact that although menstrual cups are proven to be cost-effective, various factors continue to influence their knowledge, attitude, and practice. This research is a need of the hour, as it sheds light on the factors which may promote the usage and acceptance of menstrual cups, which could become beneficial, especially in urban slums and rural areas where menstrual hygiene is seldom maintained due to various cultural factors.

A major limitation of the study is its cross-sectional design, which makes establishing causal relationships between variables challenging. Additionally, given the personal and potentially sensitive nature of menstrual cup usage, participants may be influenced by societal norms or expectations when responding. This introduces a risk of social desirability bias, which could impact the accuracy of self-reported data. Future studies could adopt a longitudinal or interventional study design, which would allow for the assessment of temporality and help establish causal relationships between awareness, attitudes, and the sustained adoption of menstrual cups, particularly in response to targeted health promotion interventions.

## Conclusions

The present study highlights that a significant proportion of female undergraduate medical students are aware of menstrual cups, with social media being the primary source of information. However, awareness does not necessarily mean acceptance and usage, indicating barriers such as a lack of in-depth knowledge, concerns regarding safety and comfort, and cultural or social apprehensions.

In order to bridge the existing gaps, targeted interventions such as educational initiatives, community workshops, and awareness campaigns are essential. Providing clear, evidence-based information about the proper usage and long-term advantages of menstrual cups can help overcome the common misconceptions. Peer support and advocacy by the young medical students who serve as vital links between health systems and the community can help establish menstrual cup usage as a socially accepted, eco-friendly, and economical option for menstrual hygiene practices.

## Appendices

Appendix A 

**Table 6:** Questionaries guide

Questionnaire	
Have you heard of or knew about menstrual cups? Yes/No	
1)	Participant ID
2)	Age
	18 years to ≤21 years
	≥ 22 years to 25 years
3)	Marital status
	Never married
	Married
4)	Educational qualification of the family head: (SES)
	Professions of honour/postgraduate
	Graduate/diploma
	High school
	Middle school
	Primary school
	Illiterate
5)	Occupation of the family head
	Professionals/Manager/Agriculture
	Technicians/skilled worker
	Clerks/shopkeepers/market sales worker/semi-skilled worker
	Unskilled worker
	Unemployed
6)	Monthly income of the family head
	>19575
	9788-19574
	7323-9787
	4894-7322
	2936-4893
	980-2935
	<978
7)	How did you know about the usage of menstrual cup? Family member/friend/social media
8)	What is the menstrual cup made of? Silicone/Rubber & latex/Plastic/Don’t know
9)	Do any of your family members use menstrual cups? Yes/No
10)	Do any of your friends use menstrual cups? Yes/No
11)	Do you find that the menstrual cups are easily available? Yes/No
12)	Do you suffer from any menstrual problems? Yes/No
13)	Is buying sanitary napkin a concern for you? Yes/No
14)	Is disposing sanitary napkin a concern for you? Yes/No
15)	Have you been advised by a doctor on problems related to menstrual hygiene? Yes/No
16)	Do you engage in any sports activities? Yes/No
17)	Do you feel menstrual cups are difficult to use? Yes/No
	Knowledge Questions (Maximum score -12 Minimum score - 6)
18)	What is the recommended duration for wearing a menstrual cup before emptying it?
	a) 4-6 hours b) 8-12 hours Expected answer: 8-12 hours (2 points) 4-6 hours (1point)
19)	How should a menstrual cup be sterilized before the next menstrual cycle?
	a) Boil in water for 5-10 minutes b) Wash with regular soap and water Expected Answer: Boil in water for 5-10 minutes (2 point) Wash with regular soap and water (1 points)
20)	What is the proper way to remove a menstrual cup?
	Pull it out forcefully using the stem Pinch the base to release suction before removal Expected answer: Pinch the base to release suction before removal (2 points) Pull it out forcefully using the stem (1 points)
21)	How long can a well-maintained menstrual cup typically last before needing replacement?
	a) 1-2 years b) 5-10 years Expected answer: 5-10 years (2 points) 1-2 years (1 point)
22)	What is a common reason that women find it difficult to use a menstrual cup initially?
	a) Improper folding and insertion technique b) The cup expands before insertion Expected answer: Improper folding and insertion technique (2 points) The cup expands before insertion (1 point)
Attitude Questions (Maximum score - 12; Minimum score - 6)	
23)	Menstrual cup is more convenient than other sanitary protection Agree Disagree Expected Answer: Agree (2 Points) Disagree (1 point)
24)	Menstrual cup is cost effective Agree Disagree Expected Answer: Agree (2 Points) Disagree (1 point)
25)	Menstrual cup can be worn overnight Agree Disagree Expected Answer: Agree (2 Points) Disagree (1 point)
26)	Usage of menstrual cups can produce odour Agree Disagree Expected Answer: Disagree (2 Points) Agree (1 point)
27)	It interferes with the daily activity Agree Disagree Expected answer: Disagree (2 points) Agree (1 point)
28)	Will you recommend it to others? Agree Disagree Expected Answer: Agree (2 Points) Disagree (1 point)
Practice Questions	
29)	What sanitary product were you using before menstrual cup? Tampons Sanitary Pads Homemade cloths
30)	How do you store the menstrual cup? Cloth bags Plastic bags Air tight containers
31)	Have you experienced any leakage? Yes Sometimes No
32)	Have you encountered any side effects? Pain Rashes/dryness UTI None
33)	After each use, do you wash the menstrual cup? Yes Sometimes No
34)	How often do you have to empty? once/day b) 2-4 times/day c) >5 times/day
